# Identification and characterization of cresol degrading *Pseudomonas monteilii* strain SHY from Soil samples

**DOI:** 10.6026/97320630014455

**Published:** 2018-10-03

**Authors:** Shainy Nhattuketty Krishnan, Anuraj Nayarisseri, Usha Rajamanickam

**Affiliations:** 1 Department of Microbiology, Karpagam University, Karpagam academy of higher education, Eachinary, Coimbatore - 641 021, Tamilnadu, India; 2 Department of Microbiology, Safi center for scientific research, Vazhayoor East, Malappuram- 673 633, Kerala, India; 3 In silico Research Laboratory, Eminent Biosciences, Vijaynagar, Indore - 452010, Madhya Pradesh, India; 4 Bioinformatics Research Laboratory, LeGene Biosciences Pvt Ltd, Indore - 452010, Madhya Pradesh, India

**Keywords:** Cresol degrading bacteria, *Pseudomonas monteilii*, 16S rRNA Gene Sequencing

## Abstract

Cresol is an organic pollutant discharged by pharmaceutical, pesticide, coal and gasification industries causing severe organ failure in humans. Therefore, it is of interest to isolate microbes from contaminated site for degrading cresol. We isolated a strain (CR-13) that survives at 5000ppm cresol with about 80 percent cresol degradation ability. Immobilized cells showed >99 percent degradation at high concentration of cresol. The 16S rRNA sequence (accession number: MF278026) deposited in GenBank was used for phylogenetic tree analysis and the strain was grouped under Pseudomonas monteilii. The isolated cresol degrading strain was subsequently named as Pseudomonas monteilii SHY.

## Background

Cresol is a phenol derivative that has intense applications in coal gasification, plastic, pharmaceutical, petro-chemical and dye industries [1]-[3]. Cresol is a harmful pollutant at low concentration of 12mg/L and has the potential to damage body organs including heart, kidney, liver, eyes, etc. based on the exposure time and amount [3]. The usual cresol degradation procedure involves expensive photo-catalytic and electro-chemical oxidation processes [4]. The growing concern for degrading cresols has opened an option for bioremediation. Bioremediation uses plants and microorganisms to degrade which in turn is non-hazardous and eco-friendly. Many species of Acinetobacter, Alcaligenes, Arthrobacter, Aspergillus, Bacillus, Candida, Ewingella, Pseudomonas, Ralstonia, Rhodococcus, Sphingomonas and Streptomyces have been used in bioremediation [1], [5]-[11].

Cresol has a phenolic ring with hydroxyl group that is degraded using various enzymes such as hydroxylase and dioxygenase that are present in the microorganisms. These microorganisms basically use enzymes to convert the compound into energy source [12]. The action of these enzymes breaks down the complex into consumable form for survival of microorganism. Initially, hydroxylase act on the phenolic ring forming catechol followed by oxidation resulting into ring opening [13], [14]. The oxidation is basically of two types ortho and meta, where, two different types of enzymes are involved, catechol 1,3 dioxygenase and catechol 2,3 dioxygenase, acting respectively [10], [15]. The ortho-oxidation pathway in degradation process is also referred to as beta-ketoadipate pathway [2],[16].

Cell immobilization has been applied to preserve the cells for a longer duration without loss of cell activity [17]. The biocompatible matrix is generally used for the purpose which gives the microbial system support for growth, prevention from toxicity, ease of scale-up i.e. use in fermenters or bioreactors, reduces escape of microbes into the effluent stream [5]. Calcium alginate is quite known and used for cell entrapment, whereas, agar had been sparsely used. Various strains of Arthrobacter, Acinetobacter, Pseudomonas, Ralstonia, fungus have been immobilized on calcium alginate beads [5], [7]. Calcium alginate is also applied with gelatin to create a strong gel matrix known as hydrogel. These hydrogel are non-toxic, biocompatible and iodegradable due to biological origin with high adsorption potential [18]. They are mostly used in pharmaceutical industries for drug delivery, contact lenses, wund dressing, etc. Hydrogels have been used as matrix for growing anchorage-dependent cells in tissue culture because they are soft and easily penetrable for the cells to adhere over the surface providing mechanical support to grow and proliferate. Its application in cell entrapment is known [18]. Other polymeric matrices used for cell immobilization include polyurethane foam. These are chemical based polymers with high porosity, mechanical strength and surface area for interaction. The foams are easy for application in stirred tank reactors for large scale production [19].

Degradation of cresol using a potential microorganism screened from a petrochemical contaminated site is of interest. A series of degradation assay were performed to isolate the prospective microbial species. Phylogenetic assessment along with biochemical characterization aided in the identification of bacterium. The study was followed by bacterial immobilization using calcium alginate, agar beads, polyurethane foam and calcium alginate gelatin beads. The physical and chemical parameters for the suitable matrix were optimized and compared with the optimized conditions used for degradation of cresol by free cells.

## Methodology

### Screening and isolation

The microbial consortium was isolated from various petroleum-contaminated sites including petrol pumps, automobile workshops and petroleum reservoirs, in and around Calicut, Kerala, India. The soil samples were collected in autoclaved bottles and were stored at 4°C until use. The soil was diluted with 0.85% saline at 1% concentration and kept for 15 minutes to get dissolved. The well-dissolved 1%soil-saline solution, thus, obtained was inoculated in to nutrient broth for growth of microorganisms for a period of 24 hours at ambient temperature [20],[21]. The temperature and period of incubation supports the growth of various bacterial species acclimatized to the petroleum contaminated environment.

Preparing specific growth medium isolated the microbial species with cresol degradation ability. The well-known cresol degrader, Pseudomonas putida, was grown on Minimal mineral medium (MMM) without carbon source and was used as a negative control, whereas, for positive control, P. putida was grown on MMM with 100ppm of each cresol isomers, p-, o- and m- cresol, simultaneously, as carbon source [22][22]. The test to be analyzed for degradation efficiency was also grown on each of the cresol isomer supplemented in MMM as carbon source at a concentration of 100 ppm. The samples were incubated for 24 hours at 37°C and optical density was calculated at 550 nm using UV-Visible spectrophotometer. Based on maximum optical density achieved with one of the cresol isomer as carbon source, that particular cresol was selected for further studies.

After the incubation period of 24 hours, survivor microorganisms were transferred to MMM supplemented with a higher dose of selected cresol isomer i.e. 500ppm. On completion of 24 hours incubation, dilution of the grown culture was prepared from 10^-1^ to 10^-10^ using distilled water. These dilutions were spread on to solid medium prepared using MMM with 2% agar and 500ppm cresol. 50μl of each dilution was spread on to the agar medium in triplicates at same physical conditions [23].

### Acclimatization

The bacteria that survived on agar plates were cultured in the same medium with cresol as carbon source (500ppm). The cultures were then subjected to higher cresol concentrations varying from 500ppm to 1200ppm. The cultured organism with maximum survivability was selected for further study.

### Growth profile

The growth profile of the bacterium was studied using O.D. at 550 nm. Also, growth profile of consortium during screening process was assessed by UV-Visible spectrophotometer.

### Cresol concentration assessment

The biomass is separated from the sample by centrifugation at 8000 rpm for 10 minutes. The amount of remnant cresol in the sample was tested by 4-amino antipyrine assay. The assay is performed by treating sample containing cresol with 4-amino antipyrine in presence of potassium ferricyanide as oxidizing agent at pH 10.00. The solution results into a stable antipyrine dye with reddish-brown color. The intensity of color is proportional to the amount of cresol concentration. UV-Visible spectrophotometer is used for measuring optical density at 506 nm that results based on color intensity. The colorimetric assay can be used for concentration calculation by plotting standard curve with known concentrations (concentration used in the study was 100 to 1000μg/L). The protocol is the modification of procedure suggested by [24]-[27].

### Immobilization of isolated bacterium

The matrices used for immobilization of the organism were calcium alginate beads, agar beads, polyurethane foam, and calcium alginate with gelatin [5], [18], [19].

### Calcium alginate entrapment

3% sodium alginate solution is prepared using growth medium and 25g of freshly grown biomass was added and mixed by eliminating the air bubbles. The cross linking was carried out by dropping (preferably using syringe) the prepared solution into stirring 100 ml of calcium chloride prepared in 0.05M concentrations. Calcium chloride is a cross linking solution that forms a gel on contact with sodium alginate. Beads were left for drying at room temperature. The dried bead were washed again with the cross linking solution and redried for use [28].

### Agar beads

4 percent agar solution was heated to get clear solution and was cooled down to 40°C. Equal amount of biomass was added to make final concentration of agar to 2% using syringe/pipette the suspension of cells and agar was pipette onto a clean petridish to form cell beads [5].

### Polyurethane foam entrapment

The polyurethane foams were commercially availed and autoclaved before immobilization. The heat stable foams were cut into pieces of 2X2X2 cm3 and kept in 100 ml of optimized growth medium. 10g of wet cell biomass was suspended in the medium and kept for incubation at optimized temperature for 24 hours. The cells gradually start growing on the foam matrix [29],[30].

### Calcium alginate cross-linked with gelatin entrapment

The entrapment method involved preparation of sodium alginate with gelatin in 2:1 ratio. The procedure is same as mentioned for sole calcium alginate bead preparation. The beads are left in stirring calcium chloride solution for saturation for 48-72 hours. The beads are dried for use [18].

### Cresol degradation study using immobilized agar beads

The two different cresol concentrations were prepared, namely 1000 and 2000 mg/L. The entrapped cells were placed in the growth medium supplemented with cresol for 24 hours. The biomass separated by centrifugation followed by evaluation of remnant cresol concentration, which was carried out by 4-amino antipyrine assay. Also, for better cresol degradation the optimization of physio-chemical parameters like pH, temperature, time, initial cresol concentration and bead size, was performed.

### Biomass estimation

The cell mass was calculated in terms of cell protein, where Lowry's protein estimation assay was used to determine the cell protein. The known quantity of the culture broth was centrifuged at 6000 rpm and cell pellet was suspended in 3.5 ml of distilled water. 0.5 ml of 20% NaOH was added and boiled in water bath for 10 minutes. The cooled solution was used subjected to Lowry's assay [31].

In case of free cells, the above mentioned protocol was used, though for agar beads, the beads have to be blotted to remove the medium and macerated in 20% NaOH solution to digest the cells. Thereafter, supernatant was subjected to Lowry's assay.

### Phenotypic characterization

The phenotypic assessment was carried out through 16s rRNA sequencing [32]-[35]. The sequencing was performed using 16S rRNA forward and reverse primers, respectively:

397: CDGGHCTANCAVATGCWAGTS

398: GMCGGRTGKGTACHAGGY

The obtained 16S rRNA sequence for respective organism, the phylogeny was assessed by similarity search against the available database and microorganism was determined.

## Results and Discussion

### Screening isolation and characterization

**Figure 1:**
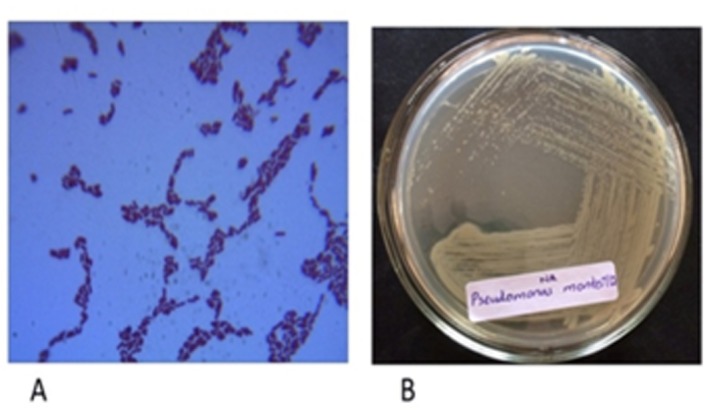
: Pseudomonas monteilii strain. (a) Gram stained smear of the isolate (Gram negative), (b) Pseudomonas monteilii growing on nutrient agar

**Figure 2:**
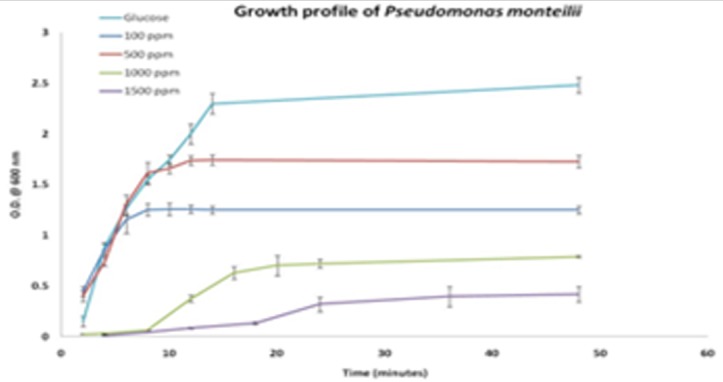
Growth profile of CR-13 at varying cresol concentration at optimized conditions (pH at 6.8, temperature at 28 degree Celcius and MMM 3 medium) is shown.

The screening for strains in samples from 30 different petroleum contaminated sites was completed. The cell growth in mineral medium on supplementing with cresol as carbon source yielded 15 competent strains able to survive on cresol. Also, o-cresol isomer was selected to carry out further experiments, as cell growth was found maximum in terms of optical density, compared to p- and m- cresol isomers. The maximum degradation efficiency of each strain was analyzed by sub-culturing the cells on to mineral medium with varying concentrations of o-cresol from 500ppm to 1200ppm. It was observed that CR-13 i.e. strain no. 13 was fittest and compatible with even high cresol concentration. Thus, CR-13 was selected for the remaining study. The screening and isolation of CR-13 was followed by morphological and biochemical characterization and the bacteria were identified as Pseudomonas monteilii [35]. The negatively gram stained rods have been clearly depicted in Figure 1A with circular smooth and wet colonies shown in Figure 1B. At the optimized conditions, the effect of initial cresol concentration was analyzed by varying cresol amount from 500ppm to 1500ppm. An increasing trend in cell growth is very well observed with increasing incubation period with a simultaneous decrease in the cresol concentration over the period of time. The best observation to be reported is the degradation ability of the strain even when cresol was present at a very high concentration of 1500ppm. This clearly showcases the benefits and necessity of optimized conditions. Similar study was performed on Pseudomonas putida to test in influence of changing phenol concentration [36]. Figure 2 depicts the corresponding graphical data at varying cresol concentration (Table 1) .

### Immobilization of Pseudomonas monteilii strain:

**Figure 3:**
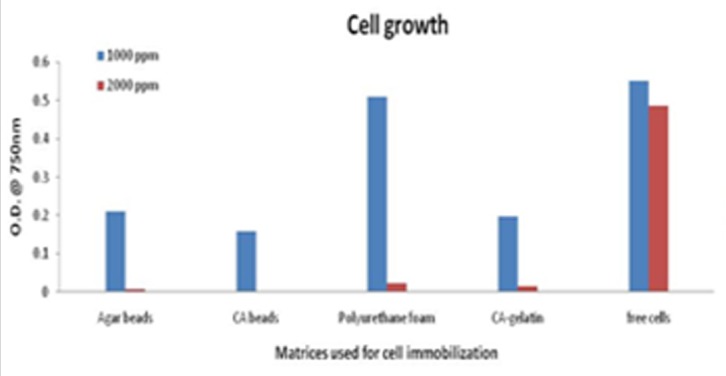
Cells immobilized on different matrices showing growth at 1000 and 2000 ppm cresol concentration, respectively is shown

Cell immobilization has various benefits. It he cells can be used for therapeutic and analytical applications [5]. The matrices used in the study were calcium alginate, agar, polyurethane foams and calcium alginate with gelatin (Figure 12). The cell entrapment was successfully achieved by each of the techniques and has been depicted in Figure 3. The efficiency of immobilized cells was further evaluated and compared with the efficiency of free cells. The experiments were performed to check the influence caused by cell entrapment.

**Figure 4:**
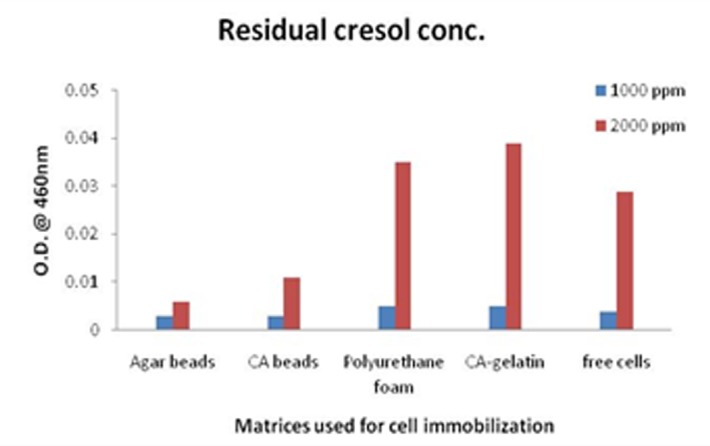
Cells immobilized on different matrices showing remnant cresol concentration, where initial concentrations were at 1000 and 2000 ppm, respectively

The cell growth study conducted at 1000 and 2000ppm cresol concentrations resulted in maximum growth of free cells followed by cell growth in polyurethane foam, agar beads, calcium alginate-gelatin beads and calcium alginate beads. The cell growth is completely depending on the supply of nutrients inside the matrices. The matrices are supposed to possess some resistance towards mass transfer thus, results in less growth. But, the cell growth was comparable with free cells, though free cells have shelf life of 3 days. Immobilization increased the storage life of cells from 3 to 45 days. A comparative analysis between calcium alginate and agar beads was conducted using Pseudomonas species by Ahamad * et al. * [5]. There have been a few studies on comparison between immobilization matrices and another study that adds to the list was performed by Mollaei et al. [37]. The minimum cresol concentration after storage period was observed in case of agar beads and has been shown in Figure 4 using Table 2 and Table 3. Thus, agar beads were further considered to carry out the optimization study.

### Parameter optimization for immobilized cells:

**Figure 5:**
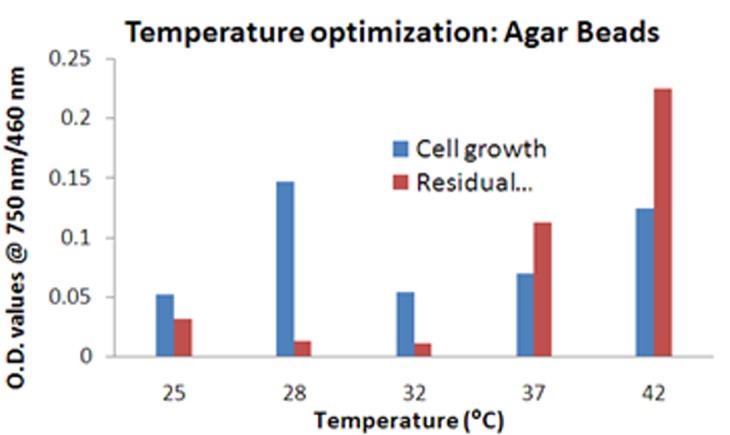
Graph depicting the cell growth on agar bead along with degraded cresol at varying temperature

**Figure 6:**
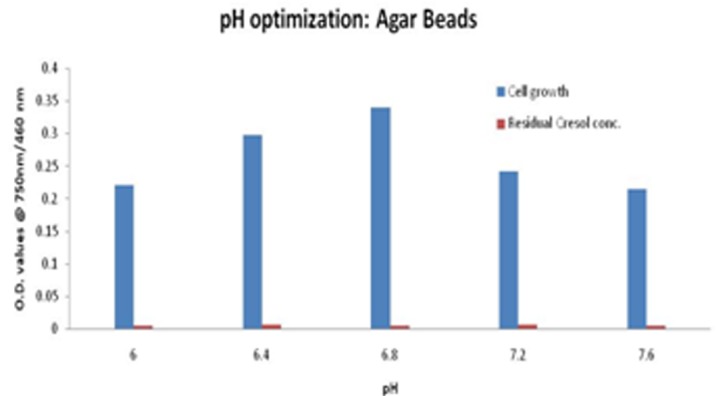
Graph depicting the cell growth on agar bead along with degraded cresol at varying pH

**Figure 7:**
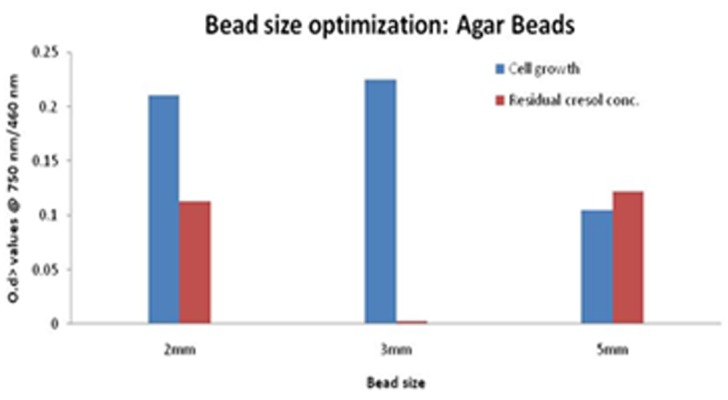
Graph depicting the cell growth on agar bead along with degraded cresol for varying agar bead size

The growth of immobilized cells on matrices can be enhanced further by optimization. The conditions optimized were pH, temperature and bead size. At temperature range from 20 to 40°C, highest cell viability was ascertained at 28°C. The OD values at different time points have been mentioned in Figure 5. The optimized pH for free cells was found to be 6.8 ± 0.2. Thus, for immobilized cells the pH range was set from 6.0 - 7.6. The cell growth increased on going from pH 6.0 to 6.8, and then decreased gradually. Although the proliferated cells were comparable at each point, thus, cresol amount was degraded to almost negligible in each case accounting to a lesser OD value, shown in Figure 6. Maximum degradation was obtained at pH = 6.8. Bead size was another factor that seemed to influence the cresol degradation. On varying bead size from 2 - 5 mm diameter, 3mm was found to be optimum size that provides less mass transfer resistance and better degradation as seen in Figure 7 showing less OD in respective case.

Cell growth in terms of cell protein was found more in case of free cells in comparison to immobilized cells. The cell protein estimation basically reveals the amount of biomass including viable and non-viable cells. Free cells have an early onset of growth, whereas, the growth may be delayed in immobilized matrix due to less contact of cells with the medium. Thus, saving the direct contact of cells with medium possess both advantages and disadvantages. The benefits account to the preserved cell potential for degradation of cresol even at higher concentration. On the other hand, the delayed cell growth in the initial cell growth stages results in cresol degradation over extended time frame. The cell growth in free cells and immobilized matrix can be seen in Table 4 and Table 5.

**Figure 8:**
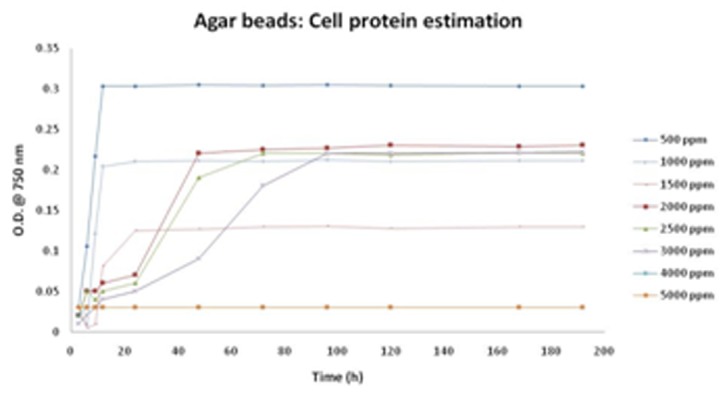
Cell growth on Agar bead as matrix

**Figure 9:**
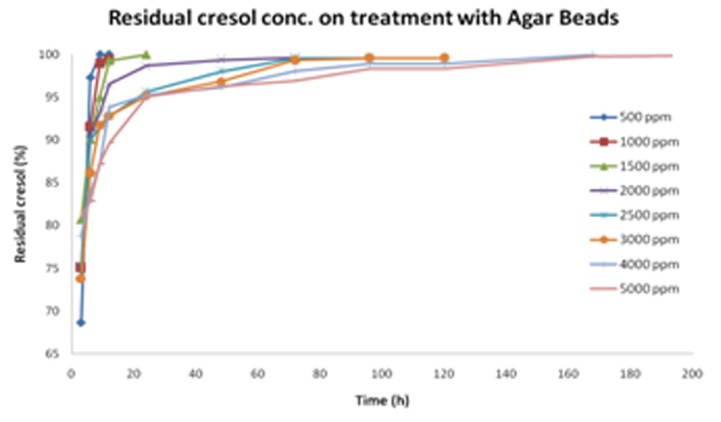
Cresol degradation by cell immobilized on Agar beads

**Figure 10:**
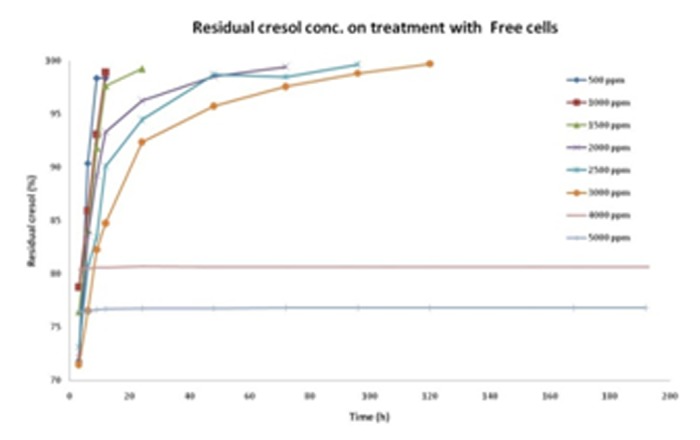
Cresol degradation by free cells

Based on the bead size, agar beads were further tested as the matrix to grow cell and degrade cresol. The study conducted on Agar beads gave an enhanced resistance and degradation potential to cells against cresol. The cells were able to survive well even at high cresol concentration of 5000ppm. Figure 8 depicts the corresponding graph for growth profile of CR-13 in agar beads and free cells, respectively. Notably, free cells were able to survive up to 3000ppm cresol concentration. Also, cresol degradation with respect to time has been graphically presented in Figure 9 and Figure 10 for cells immobilized on agar beads and free cells, respectively.

**Figure 11:**
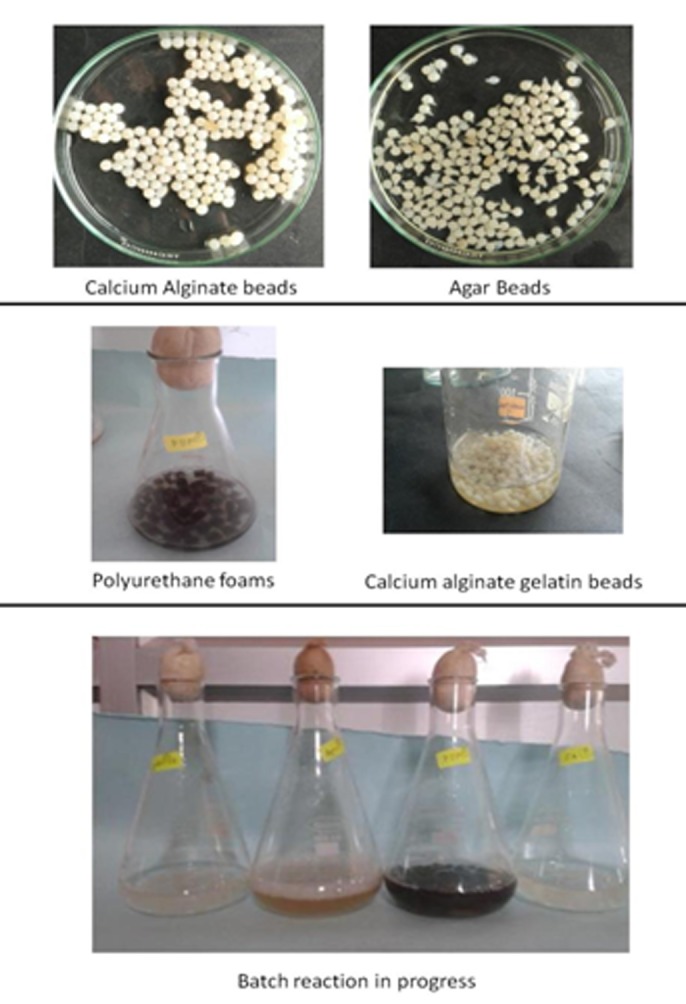
The immobilization matrices used in the study

The optimized conditions for immobilized cells on different agar beads (Figure 11) were further used for cresol degradation and compared simultaneously with free cells. Table 6 gives a detailed outlay of the variation in residual concentrations of cresol over the time. The free cells have potential to degrade more than agar beads when the cresol concentration is <3000ppm due to easy contact with cresol as carbon source (Figure 9). Ongoing to the higher cresol amount, agar beads have shown better potential which may be due to the protecting layer provided by agar. The direct contact of cresol to the cells was somehow lesser in case of immobilization that resulted in cresol utilization. Cresol being hazardous chemical, might cause cell death on direct exposure leading to ~80% degradation at >3000ppm cresol amount.

### Phylogeny assessment

The strain was subjected to DNA isolation using phenol-chloroform method. PCR amplification of the 16S rRNA of the isolated DNA was carried out using protocol mentioned in [21]. Amplified product of the DNA was used for 16S rRNA sequencing using Sanger dideoxy Sequencing. Both the forward and reverse sequence obtained from the trace file, further assembles using DNA Baser [38]. The assembled sequence obtained in FASTA format shown below was used for the further Bioinformatics analysis [39], [44].

**Figure 12:**
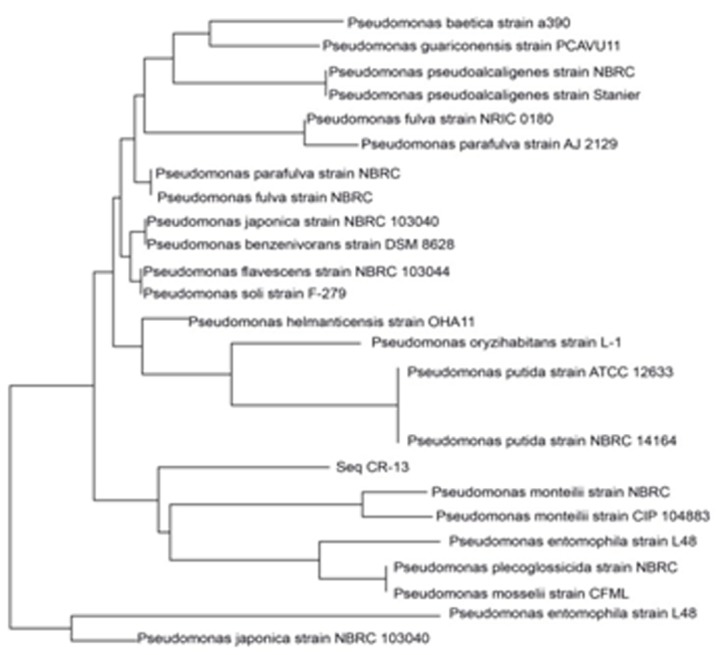
Phylogenetic affiliation of Sequence of Pseudomonas monteilii SHYagainst all the other species of Pseudomonas monteilii

The CR-13 sequence was used for pairwise alignment using Blast against 16S ribosomal RNA Database in NCBI. All the similar sequences were retrieved from NCBI and the same was used for constructing the phylogenetic Tree (Figure 12) using Mega [45]. The sequence of sample CR-13 aligned against other species was concluded to be a novel strain, further which were named Pseudomonas monteilii strain SHY. The sequence of the novel isolate was deposited in NCBI Database using Accession number: MF278026.

### Elucidation of RNA secondary Structure of Pseudomonas monteilii strain SHY

**Figure 13:**
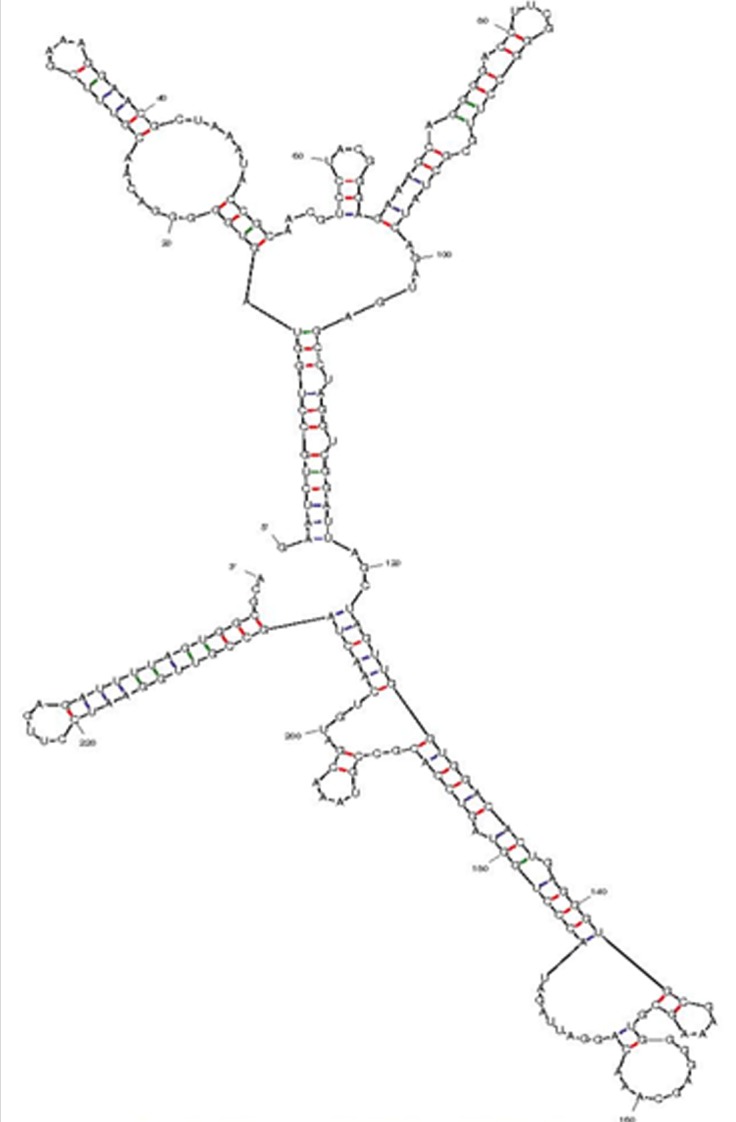
RNA Secondary Structure of Pseudomonas monteilii strain SHY elucidated by UNAFOLD

RNA secondary structure prediction [46],[47] of Pseudomonas monteilii strain SHY is relevant. The 16S rRNA sequence of Pseudomonas monteilii strain SHY was formatted n FASTA to deduce the secondary structure of RNA using UNAFOLD (Figure 13) in Linux Platform [47]. .ct file and .reg file which were obtained from the UNAFOLD was further used to analyse the secondary structure of Pseudomonas monteilii strain SHY. The result shows that the free energy of the secondary structure of rRNA was DG, ∆G = -275.70 kcal/mol which found to be a stable structure. The thermodynamics result from each base wise of the dataset shows the average of external closing pair helix External loop-4.20, Helix Multi-loop-9.60, Helix Bulge loop-4.90, Helix Interior loop-9.10of DG -62.10 kcal/mol respectively.

## Conclusion

We report the isolation and optimization of Pseudomonas monteilii strain SHY (CR-13) for cresol degradation. The isolated cresol-degrading microbe was characterized by the phylogenetic tree analysis of 16S rRNA sequence. Cresol degradation by Pseudomonas monteilii strain SHY was optimized at varying temperatures, pH and media for varying concentration of cresol. The microbe immobilized in agar beads improved cresol degradation efficiency.

## Figures and Tables

**Table 1:** OD values (550 nm) for growth profile of CR-13 at optimized pH, temperature and growth medium with varying cresol concentration

OD in presence of Glucose (control)	Cresol Concentration (ppm) with respective OD values			
	100	500	1000	1500
0.15 ± 0.05	0.446667 ± 0.050332	0.403333 ± 0.056862	0.024 ± 0.005292	0.013333 ± 0.005774
0.89 ± 0.036	0.86 ± 0.052915	0.74 ± 0.045826	0.032667 ± 0.006429	0.045667 ± 0.005132
1.256667 ± 0.051	1.153333 ± 0.136137	1.3 ± 0.1	0.067 ± 0.002646	0.086333 ± 0.005508
1.546667 ± 0.05	1.253333 ± 0.061101	1.616667 ± 0.104083	0.375 ± 0.035	0.134667 ± 0.013614
1.743333 ± 0.051	1.256667 ± 0.060277	1.66 ± 0.052915	0.63 ± 0.060828	0.32 ± 0.072111
2 ± 0.1	1.256667 ± 0.040415	1.74 ± 0.045826	0.703333 ± 0.100167	0.393333 ± 0.100664
2.3 ± 0.1	1.25 ± 0.036056	1.743333 ± 0.051316	0.723333 ± 0.040415	0.416667 ± 0.076376
2.483333 ± 0.076	1.25 ± 0.036056	1.73 ± 0.060828	0.79 ± 0.01	0.013333 ± 0.005774

**Table 2:** Residual cresol concentration (%) in presence of CR-13 immobilized on Agar Beads

	Initial cresol concentration (ppm)							
	500	1000	1500	2000	2500	3000	4000	5000
Time (h)	Residual cresol concentration (%) with respect to time							
3	68.6464	75.0342	80.70213	74.3798	75.3636	73.8078	78.76345	81.15296
6	97.3096	91.489	90.0796	90.7019	90.01368	86.10467	83.7397	82.79844
9	99.9636	98.9202	95.03373	93.0905	91.49992	91.76653	87.12355	87.25716
12	99.9636	99.451	99.28013	96.5407	92.77384	92.82813	93.8249	89.64576
24			99.98787	98.6639	95.64016	95.12827	95.2846	95.05992
48				99.3274	97.97568	96.80913	96.14715	96.28076
72				99.5928	99.56808	99.2862	98.00495	96.91772
96					99.56808	99.5516	98.8675	98.2978
120					99.56808	99.5516	98.8675	98.2978
168							99.9291	99.73096
192								99.78404

**Table 3:** Residual cresol concentration (%) in presence of CR-13 free cells

	Initial cresol concentration (ppm)							
	500	1000	1500	2000	2500	3000	4000	5000
Time (h)	Residual cresol concentration (%) with respect to time							
3	71.8312	78.7498	76.45573	72.1239	73.13424	71.50767	80.46292	76.50755
6	90.4092	85.9156	84.06387	83.6688	80.6716	76.55027	80.56908	76.50755
9	98.3712	93.0814	91.84893	89.2422	83.43176	82.3006	80.62216	76.64025
12	98.3712	98.9202	97.68773	93.3559	90.11984	84.77767	80.62216	76.7066
24			99.28013	96.2753	94.4724	92.3858	80.72832	76.77295
48				98.5312	98.7188	95.74753	80.67524	76.77295
72				99.4601	98.50648	97.60533	80.67524	76.8393
96					99.67424	98.84387	80.67524	76.8393
120						99.72853	80.67524	76.8393
168							80.67524	76.8393
192								76.8393

**Table 4:** OD values taken at 600 nm for cell protein estimation using Lowry's assay for agar beads immobilized CR-13 cells

	Initial cresol concentration (ppm)							
Time (h)	500	1000	1500	2000	2500	3000	4000	5000
	OD at 600 nm with respect to time							
3	0.03	0.02	0.03	0.02	0.02	0.01	0.02	0.03
6	0.106	0.009	0.005	0.05	0.05	0.02	0.03	0.03
9	0.217	0.121	0.009	0.05	0.04	0.03	0.03	0.03
12	0.303	0.204	0.081	0.06	0.05	0.04	0.03	0.03
24	0.303	0.21	0.125	0.07	0.06	0.05	0.03	0.03
48	0.305	0.211	0.127	0.22	0.19	0.09	0.03	0.03
72	0.304	0.21	0.129	0.225	0.22	0.18	0.03	0.03
96	0.305	0.212	0.13	0.227	0.22	0.22	0.03	0.03
120	0.304	0.21	0.128	0.23	0.219	0.22	0.03	0.03
168	0.303	0.211	0.129	0.229	0.221	0.221	0.03	0.03
192	0.303	0.211	0.129	0.23	0.22	0.222	0.03	0.03

**Table 5:** OD values (600 nm) for cell protein estimation using Lowry's assay for CR-13 free cells

	Initial cresol concentration (ppm)							
Time (h)	500	1000	1500	2000	2500	3000	4000	5000
	OD at 600 nm with respect to time							
3	0.244	0.197	0.191	0.178	0.171	0.155	0.149	0.132
6	0.422	0.331	0.269	0.225	0.204	0.182	0.15	0.135
9	0.542	0.461	0.372	0.298	0.254	0.216	0.155	0.14
12	0.542	0.516	0.469	0.384	0.304	0.263	0.16	0.149
24	0.55	0.553	0.532	0.475	0.382	0.322	0.17	0.155
48	0.56	0.554	0.54	0.532	0.486	0.431	0.17	0.157
72	0.55	0.56	0.55	0.54	0.52	0.494	0.173	0.16
96	0.56	0.55	0.55	0.55	0.53	0.499	0.172	0.167
120	0.55	0.554	0.553	0.552	0.535	0.51	0.17	0.165
168	0.55	0.55	0.554	0.553	0.539	0.512	0.17	0.16
192	0.55	0.55	0.55	0.552	0.53	0.52	0.169	0.16

**Table 6:** Top BLAST resultsfor 16S ribosomal RNA (Bacteria and Archaea)

Query acc	subjacc	% iden	align len	q. start	q. end	s. start	s. end	e-value	bit score
seq CR-13	NR_116172.1	98.919	740	1	739	113	845	0	1315
seq CR-13	NR_114224.1	98.784	740	1	739	95	827	0	1310
seq CR-13	NR_112073.1	98.784	740	1	739	95	827	0	1310
seq CR-13	NR_024910.1	98.784	740	1	739	106	838	0	1310
seq CR-13	NR_102854.1	98.649	740	1	739	115	847	0	1304
seq CR-13	NR_114226.1	98.649	740	1	739	95	827	0	1304
seq CR-13	NR_024662.1	98.649	740	1	739	115	847	0	1304
seq CR-13	NR_024924.1	98.514	740	1	739	106	838	0	1299
seq CR-13	NR_115336.1	98.108	740	1	739	101	833	0	1290
seq CR-13	NR_114192.1	97.838	740	1	739	95	827	0	1273
seq CR-13	NR_113651.1	97.838	740	1	739	95	827	0	1271
seq CR-13	NR_114479.1	97.838	740	1	739	111	843	0	1271
seq CR-13	NR_025881.1	97.703	740	1	739	113	845	0	1266
seq CR-13	NR_126220.1	97.568	740	1	739	101	833	0	1260
seq CR-13	NR_134794.1	97.568	740	1	739	58	790	0	1260
seq CR-13	NR_114195.1	97.568	740	1	739	95	827	0	1260
seq CR-13	NR_116904.1	97.568	740	1	739	107	839	0	1260
seq CR-13	NR_040992.1	97.568	740	1	739	115	847	0	1260
seq CR-13	NR_043421.1	97.568	740	1	739	113	845	0	1260
seq CR-13	NR_113855.1	97.432	740	1	739	95	827	0	1254
seq CR-13	NR_113856.1	97.432	740	1	739	95	827	0	1254
seq CR-13	NR_040859.1	97.432	740	1	739	107	839	0	1254
seq CR-13	NR_104278.1	97.432	740	1	739	107	839	0	1254
seq CR-13	NR_037000.1	97.432	740	1	739	95	827	0	1254
seq CR-13	NR_113653.1	97.297	740	1	739	95	827	0	1253
